# Immunomodulatory Probiotics in Psoriasis: Effects on the Gut–Skin–Immune Crosstalk and Systemic Inflammation: Immunomodulatory Probiotics and Psoriasis Disease

**DOI:** 10.1155/jnme/7365660

**Published:** 2026-11-01

**Authors:** Ahmed Hussein Hasan Alshihmani, Mahmoud Mahmoudi, Ramiar Kamal Kheder, Maryam Ahmadi-Khorram, Afsane Fadaee, Dariush Haghmorad, Mohammad Hossein Shams, Seyed-Alireza Esmaeili

**Affiliations:** ^1^ Immunology Research Center Mashhad University of Medical Sciences Mashhad Iran mums.ac.ir; ^2^ Immunology Department Faculty of Medicine Mashhad University of Medical Sciences Mashhad Iran mums.ac.ir; ^3^ Medical Laboratory Science Department College of Science University of Raparin Rania Sulaymaniyah Iraq uor.edu.krd; ^4^ Department of Medical Analysis Faculty of Applied Science Tishk International University Erbil Iraq tiu.edu.iq; ^5^ Department of Nutrition Faculty of Medicine Mashhad University of Medical Sciences Mashhad Iran mums.ac.ir; ^6^ Student Research Committee Mashhad University of Medical Sciences Mashhad Iran mums.ac.ir; ^7^ Department of Immunology School of Medicine Semnan University of Medical Sciences Semnan Iran semums.ac.ir; ^8^ Hepatitis Research Center and Department of Medical Immunology School of Medicine Lorestan University of Medical Sciences Khorramabad Iran lums.ac.ir

**Keywords:** gut microbiota, inflammation, probiotics, psoriasis, skin health

## Abstract

Psoriasis is a chronic inflammatory skin disorder characterized by dysregulated immune responses and excessive keratinocyte proliferation, with substantial effects on patients’ quality of life. Evidence suggests that the gut microbiota and the gut–skin–immune axis may contribute to systemic inflammation and immune dysregulation associated with psoriasis. This narrative review examines the potential immunomodulatory role of probiotics in psoriasis, with particular emphasis on strain‐specific biological effects and the mechanisms linking intestinal microbial modulation to psoriasis‐related inflammation. Probiotics may influence intestinal microbial composition, epithelial barrier integrity, microbial metabolite production, and immune signaling. Clinical studies have reported potential improvements in psoriasis severity, inflammatory biomarkers, or quality of life following probiotic supplementation; however, the available evidence remains limited and heterogeneous, with substantial variation in probiotic strains, formulations, doses, treatment durations, concomitant therapies, and clinical outcomes. Unlike previous systematic reviews and meta‐analyses that primarily summarize clinical outcomes, this review integrates clinical findings with strain‐specific mechanistic and experimental evidence to provide a biologically contextualized interpretation of the potential role of probiotics in psoriasis.

**Summary**
• Significance Statement
◦ Psoriasis, a chronic immune‐mediated inflammatory skin condition, is characterized by uncontrolled proliferation and dysfunctional differentiation of keratinocytes. Emerging evidence suggests that immunomodulatory probiotics may influence inflammatory and immune pathways relevant to psoriasis through modulation of the gut microbiota and the gut–skin–immune axis. Tolerogenic probiotics may modulate immune responses through regulatory pathways by influencing the production of regulatory cytokines and proinflammatory mediators, potentially contributing to changes in skin manifestations and immune dysregulation. Further well‐controlled clinical studies are needed to clarify their therapeutic potential, optimal formulations, and long‐term safety.

## 1. Introduction

Psoriasis, a chronic inflammatory skin condition driven by genetic and environmental factors, affects approximately 1%–3% of the global population, significantly impacting physical and emotional well‐being [1, 2]. Existing treatments, such as topical agents, phototherapy, systemic immunosuppressants, and biologics, aim to reduce symptoms but often face challenges such as side effects, variable efficacy, and high costs [3, 4]. Recent research highlights the gut microbiota’s role in regulating immune function and inflammation, suggesting its influence on systemic health and skin conditions like psoriasis [5–7]. Imbalances in gut microbial communities, known as dysbiosis, can disrupt immune homeostasis, contributing to autoimmune disorders [8]. The gut microbiota interacts with the immune system through various mechanisms, including the generation of metabolites such as short‐chain fatty acids (SCFAs), which have anti‐inflammatory properties [9, 10]. Microbiota‐derived fermentation products, including SCFAs, such as butyrate, propionate, and acetate, facilitate the development and function of Treg cells. They do this by inducing the expression of Foxp3 or by stimulating dendritic cells and intestinal epithelial cells to produce retinoic acids or TGF‐β1, respectively [11]. These metabolites can enhance the differentiation of regulatory T cells (Tregs) and decrease the activity of proinflammatory T‐helper 17 cells (Th17), which are implicated in the pathogenesis of autoimmune diseases like psoriasis [10–12]. Furthermore, gut microbiota can regulate the generation of cytokines and other immune mediators, which are essential for maintaining skin health and immunological homeostasis [13]. Although previous systematic reviews and meta‐analyses have evaluated the clinical efficacy of probiotic supplementation in psoriasis, their principal focus has been on combined clinical outcomes, specifically the Psoriasis Area and Severity Index (PASI), Dermatology Life Quality Index (DLQI), and selected inflammatory biomarkers. Recent meta‐analyses have synthesized randomized controlled trials and reported potential improvements in psoriasis severity and inflammatory markers; however, substantial heterogeneity in probiotic strains, formulations, doses, treatment durations, and clinical outcomes remains [14, 15].

The present review provides a mechanism‐oriented and strain‐specific synthesis of the available evidence. Specifically, the review examines evidence from clinical and experimental studies to determine how individual probiotic strains may influence gut–skin–immune crosstalk through modulation of gut microbial composition, SCFA production, intestinal barrier integrity, dendritic‐cell signaling, and the balance between regulatory T‐cells (Tregs) and Th17 cells, with particular emphasis on the IL‐23/IL‐17 inflammatory pathway [16, 17]. Additionally, the review incorporates experimental evidence from strain‐specific animal models to help connect biological mechanisms with observed clinical findings.

## 2. Literature Search and Review Methodology

This narrative review was conducted to summarize the current evidence regarding the immunomodulatory effects and therapeutic potential of probiotics in psoriasis, with particular emphasis on clinical and experimental evidence, immune mechanisms, and the gut–skin–immune pathway. Specific attention was given to human clinical studies and experimental models investigating inflammatory mediators, immune‐cell responses, gut microbiota, skin symptoms, or psoriasis‐related signaling pathways. The literature search was conducted in PubMed/MEDLINE, Scopus, and Web of Science using combinations of terms related to psoriasis, probiotics, gut microbiota, immunomodulation, inflammation, cytokines, Treg/Th17 responses, and experimental models. The search covered publications from 2015 to 2025. In addition to database searching, the reference lists of relevant articles and reviews were manually screened to identify additional studies of potential relevance. Since the present study was designed as a narrative review rather than a systematic review, study selection was based on relevance to the review objectives and the biological context of the available evidence.

## 3. Psoriasis Pathogenesis and the Gut–Skin–Immune Crosstalk

Psoriasis is a chronic immune‐mediated inflammatory disease resulting from complex interactions among genetic susceptibility, environmental exposures, immune dysregulation, and alterations in host–microbiome interactions. The IL‐23/IL‐17 pathway represents a central inflammatory mechanism in psoriasis, leading to keratinocyte hyperproliferation and persistent cutaneous inflammation. Accumulating evidence indicates that psoriasis should not be considered solely a cutaneous disorder, as systemic inflammatory processes and associated metabolic and cardiovascular comorbidities may reflect broader immune dysregulation [18, 19].

The gut microbiota has increasingly been implicated in this systemic inflammatory network. Recent systematic reviews have demonstrated differences in gut microbial composition and diversity between individuals with psoriasis and healthy controls, although the specific microbial alterations are not entirely consistent across studies [3, 4]. These observations suggest that gut dysbiosis may be associated with psoriasis, but they do not by themselves establish a causal relationship. Importantly, recent mechanistic evidence has proposed that alterations in microbial metabolites, particularly SCFAs, together with impaired intestinal barrier integrity, may influence systemic immune homeostasis and inflammatory signaling [19–21].

The gut–skin axis is increasingly recognized as a bidirectional communication network involving intestinal microorganisms, microbial metabolites, epithelial barrier function, and host immunity. Dysbiosis and disruption of intestinal barrier integrity may increase exposure to microbial products and modify systemic immune responses, thereby influencing pathways involved in psoriasis, including the Th17/Treg balance and IL‐23/IL‐17‐mediated inflammation [19, 21]. In contrast, systemic inflammation, dietary factors, lifestyle, and pharmacological treatments may also modify the intestinal microbiome, suggesting that the relationship between intestinal dysbiosis and psoriasis is unlikely to be unidirectional [20, 22].

This emerging framework provides a biological basis for investigating microbiota‐targeted interventions, particularly probiotics. Probiotics may influence intestinal microbial composition, epithelial barrier function, microbial metabolite production, and immune signaling, thereby providing several potential mechanisms through which they could modify psoriasis‐associated inflammation [15, 19]. Recent meta‐analyses of randomized controlled trials have reported potential improvements in psoriasis severity and inflammatory markers following probiotic supplementation, but the available trials remain relatively small and heterogeneous, and effects on quality of life have not been consistently demonstrated [14, 15]. Figure 1 illustrates this axis, highlighting how gut dysbiosis may contribute to psoriasis severity through immune modulation.

**FIGURE 1:**
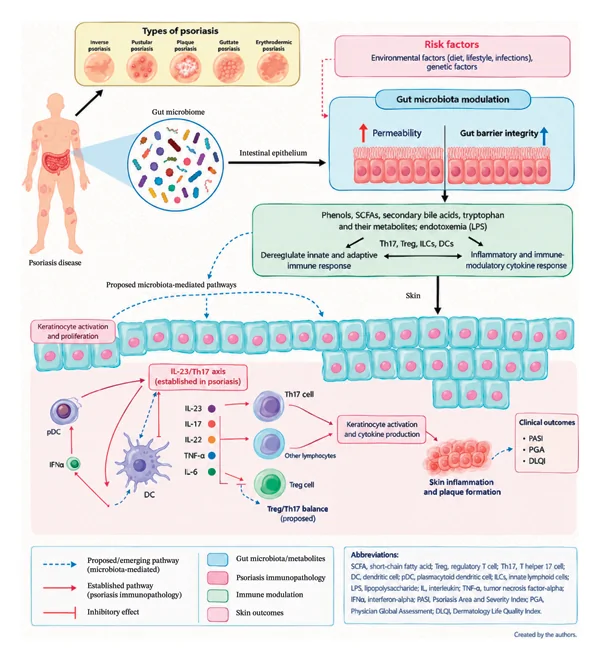
The role of gut microbiota and the gut–skin–immune axis in the pathogenesis of psoriasis. The development of psoriasis involves a complex interaction among genetic factors, environmental influences, and immune system dysregulation. Dysbiosis may impair intestinal barrier integrity and may contribute to systemic inflammatory responses and alterations in immune signaling relevant to psoriasis (the figure is created by authors).

## 4. Role of Immunomodulatory Probiotics in Psoriasis

### 4.1. Definition of Immunomodulatory Probiotics

Immunomodulatory probiotics are live microorganisms that may influence host immune responses and have been investigated as a potential adjunctive approach for inflammatory conditions such as psoriasis [17, 23]. Importantly, their biological effects are strain‐specific and cannot necessarily be extrapolated from one strain to other members of the same species or genus. Strains such as *Lactobacillus* and *Bifidobacterium* may influence intestinal barrier function, microbial composition, and immune signaling. Therefore, probiotic effects are highly strain‐ and dose‐dependent, and the available clinical evidence in psoriasis remains limited and heterogeneous. Hence, probiotics are considered an investigational therapeutic approach rather than an established adjunctive treatment [17, 24, 25].

### 4.2. Tolerogenic Probiotics

Tolerogenic probiotics refer to specific probiotic strains that have been investigated for their ability to promote immune tolerance through modulation of regulatory immune pathways [26]. Experimental studies have reported that certain strains can influence regulatory T‐cells (Tregs), tolerogenic dendritic cells, and cytokine profiles, including increased production of regulatory cytokines such as IL‐10 and TGF‐β and reduced production of proinflammatory mediators such as IL‐17 and TNF‐α. However, these effects are strain‐specific and should not be generalized to all *Lactobacillus* or *Bifidobacterium* species [25–28]. Therefore, specific probiotic strains have been reported to modulate Treg/Th17‐related pathways in experimental or disease‐specific models; however, they do not directly establish therapeutic efficacy in psoriasis [17, 28, 29].

Notably, much of the mechanistic evidence supporting the concept of tolerogenic probiotics has been derived from experimental models or from other immune‐mediated diseases, including systemic lupus erythematosus and multiple sclerosis, rather than from clinical studies of psoriasis. Studies investigating *Lactobacillus* strains and tolerogenic dendritic cells or Treg/Th17 modulation provide evidence of potential immunological mechanisms but do not directly establish therapeutic efficacy in psoriasis.

In psoriasis, evidence regarding individual probiotic strains remains comparatively limited. Experimental psoriasis models and clinical studies provide more direct evidence regarding the potential effects of specific strains on psoriasis‐related inflammation and clinical manifestations.

### 4.3. Mechanism of Action of Immunomodulatory Probiotics in Improving Psoriasis

Immunomodulatory probiotics may exert immunomodulatory effects in psoriasis through multiple pathways, primarily involving the regulation of immune responses and inflammatory signaling [30]. These microorganisms interact with pattern recognition receptors (PRRs), such as toll‐like receptors (TLRs) on immune cells such as macrophages and dendritic cells, to modulate immune signaling [24, 31–33]. This interaction influences cytokine production, decreasing proinflammatory cytokines (e.g., IL‐17, TNF‐α, and IFN‐γ) while promoting anti‐inflammatory ones (e.g., IL‐10 and TGF‐β) [31, 34, 35]. Experimental studies have also reported effects on regulatory T‐cells, Th17‐related responses, intestinal barrier integrity, and microbial metabolites such as SCFAs. However, these immunological effects are not universal across probiotic strains, and the mechanisms identified for one strain should not be assumed to apply to other strains. Specific probiotic strains may enhance regulatory T‐cell (Treg) activity, which may contribute to the regulation of excessive T‐helper 17 (Th17) responses implicated in psoriasis pathogenesis. Specific probiotic strains have been reported to influence intestinal barrier integrity, which may contribute to the regulation of systemic inflammatory responses. These mechanisms may contribute to modulation of skin inflammation and immune dysregulation; however, their clinical relevance in psoriasis remains uncertain [29, 36, 37].

### 4.4. Studies and Research in This Field

#### 4.4.1. Recent Findings of Probiotics in Clinical Trials of Psoriasis

Several clinical studies have investigated the potential effects of probiotic supplementation in patients with psoriasis, with the findings that vary according to probiotic strain, formulation, dosage, treatment duration, concomitant therapy, and study design. Some trials have reported improvements in psoriasis‐related clinical outcomes, quality of life, or inflammatory and oxidative stress biomarkers, whereas other randomized studies have not demonstrated significant clinical benefits [38–41]. Probiotic supplementation containing multiple *Lactobacillus* and *Bifidobacterium* strains was associated with improvements in clinical and quality‐of‐life measures in some studies [39], whereas *Lactobacillus rhamnosus* intervention did not result in significant improvement in plaque psoriasis severity or quality‐of‐life outcomes [40]. *Bifidobacterium infantis* reduced systemic inflammation, suggesting broader immune modulation [41]. In addition, the Bacillus‐based intervention summarized in Table 1 combined probiotics with a prebiotic, making it difficult to attribute the observed effects specifically to the probiotic component. Importantly, reductions in inflammatory biomarkers are interpreted as evidence of potential biological or immunomodulatory activity and should not be considered as evidence of definitive clinical efficacy.

**TABLE 1:** Clinical studies investigating oral probiotic supplementation in patients with psoriasis.

Study	Design and sample size	Probiotic strain(s)	Route and dose	Duration	Comparator and background psoriasis treatment	Principal clinical outcomes	Biomarker/microbiome findings	Statistical significance/effect estimate	Safety/adverse events
Buhaş et al., 2023 [42]	12‐week, single‐center, open‐label, nonrandomized clinical trial; *n* = 63 (42 intervention, 21 control)	*Bacillus indicus* HU36, *Bacillus. subtilis* HU58, *Bacillus. coagulans* SC208, *Bacillus. licheniformis* SL307, *Bacillus. clausii* SC109	Oral; 2 capsules/day, each containing 2 × 10^9^ CFU; total 4 × 10^9^ CFU/day. In addition, 5 g/day prebiotic (FOS, XOS, GOS) during the last 8 weeks	12 weeks	Control: topical antipsoriatic treatment only. Intervention: same topical treatment + probiotics + prebiotic	PASI, DLQI, skin thickness improved in the intervention group	Changes in inflammatory/metabolic markers and gut microbiota were reported; microbiome analysis was available in 15/42 intervention participants	PASI and DLQI significantly decreased in the intervention group (both *p* < 0.001)	No specific adverse‐event analysis was reported
Navarro‐López et al., 2019 [38]	12‐week, randomized, double‐blind, placebo‐controlled trial; *n* = 90 randomized; approximately 45/45 allocated to probiotic/placebo	*Bifidobacterium longum* CECT 7347, *Bifidobacterium. lactis* CECT 8145, *Lactobacillus rhamnosus* CECT 8361	Oral; 1 × 10^9^ CFU/capsule, 1 capsule/day	12 weeks; 6‐month follow‐up for relapse	Placebo: maltodextrin capsule. Patients also received standard topical treatment; those with PASI ≥ 6 received betamethasone dipropionate + calcipotriol, whereas lower‐severity patients received topical mometasone	At Week 12, PASI75: 66.7% vs. 41.9% in probiotic vs placebo groups; PGA 0/1 was also more frequent in the probiotic group	Changes in gut microbiota were reported during follow‐up; inflammatory biomarkers were also assessed	PASI75: *p* < 0.05. Probiotic supplementation was also associated with a lower risk of relapse during follow‐up	No major safety signal was identified
Moludi et al., 2021 [39]	Randomized, double‐blind, placebo‐controlled clinical trial; *n* = 50, 25 probiotic and 25 placebo	*Lactobacillus acidophilus*, *Bifidobacterium bifidum*, *Bifidobacterium. lactis*, *Bifidobacterium. longum*	Oral; approximately 1.8 × 10^9^ CFU/capsule, 1 capsule twice daily	8 weeks	Maltodextrin capsules	PASI, PSS, DLQI, and BDI improved compared with placebo	Reduced hs‐CRP, IL‐6, MDA and improved total antioxidant capacity (TAC)	PASI: *p* = 0.049; PSS: *p* = 0.047; DLQI: *p* = 0.045; BDI *p* = 0.017; hs‐CRP *p* = 0.015 IL‐6 *p* = 0.050; MDA *p* = 0.013; TAC *p* = 0.030	No serious adverse events were reported
Suriano et al., 2023 [40]	Parallel, double‐blind, randomized, placebo‐controlled trial; *n* = 103 (50 probiotic, 53 placebo)	*Lactobacillus rhamnosus*	Oral liquid formulation; approximately 6 × 10^5^ CFU/mL, 5 mL/day	6 months	Placebo: identical whey‐based formulation without active probiotic. Both groups received standard‐of‐care treatment	PASI and DLQI were assessed. No significant clinical benefit attributable to probiotic supplementation was demonstrated	No major mechanistic biomarker outcome was established as the principal endpoint	In the probiotic group, PASI change was −1.58 (*p* = 0.105) and DLQI change +0.05 (*p* = 0.873); therefore, neither was statistically significant	Probiotic formulation was generally well tolerated; no major safety concern was identified
Groeger et al., 2013 [41]	Randomized, double‐blind, placebo‐controlled intervention; *n* = 26 psoriasis patients (12 probiotic, 14 placebo)	*Bifidobacterium infantis* 35,624	Oral; 1 × 10^10^ CFU/day	8 weeks	Placebo: 5 g maltodextrin/day. No concomitant immunosuppressive/probiotic treatment was permitted	No primary clinical efficacy endpoint such as PASI response was established; study primarily evaluated systemic inflammatory biomarkers	Reduced plasma CRP and TNF‐α; IL‐6 reduction was not significant in psoriasis	CRP: *p* = 0.0425 vs. placebo; TNF‐α reduction within the probiotic group *p* = 0.0269.	No major adverse‐event signal was reported

*Note:* IL‐6: Interleukin‐6, IFN‐γ: interferon gamma, IL‐10: Interleukin‐10, MDA: malondialdehyde, and LPS: lipopolysaccharide.

Abbreviations: BDI, Beck Depression Inventory; CRP, C‐reactive protein; DLQI, Dermatology Life Quality Index; HDL‐C, high‐density lipoprotein cholesterol; hs‐CRP, high‐sensitivity C‐reactive protein; LDL‐C, low‐density lipoprotein cholesterol; PASI, Psoriasis Area and Severity Index; PSS, Psoriasis Symptom Scale; SCFA, short‐chain fatty acids; TAC, total antioxidant capacity; TNF‐α, tumor necrosis factor alpha.

#### 4.4.2. Recent Findings of Probiotics in Experimental Models of Psoriasis

Experimental studies using imiquimod (IMQ)‐induced psoriasis‐like models have investigated specific probiotic strains, including *Lactobacillus pentosus* GMNL‐77 [43, 44], *Bifidobacterium adolescentis* CCFM667 [45], *Bifidobacterium breve* CCFM638(46), and other defined strains. These studies have reported strain‐dependent effects on inflammatory cytokines, the IL‐23/Th17 axis, gut microbiota, microbial metabolites, and psoriasis‐like skin manifestations. However, the findings from these animal models should be considered preclinical evidence and cannot be directly extrapolated to clinical efficacy in patients with psoriasis. Selected experimental studies addressing inflammatory cytokines, immune pathways, gut microbiota, and psoriasis‐like skin manifestations are summarized in Table 2.

**TABLE 2:** Experimental studies investigating oral probiotics in imiquimod‐induced psoriasis‐like animal models.

Study	Animal model and sample size	Probiotic strain	Route and dose	Duration	Comparator/control	Clinical or phenotypic outcomes	Immunological outcomes	Microbiome/metabolic outcomes	Statistical significance
Chen et al., 2017 [44]	Male BALB/c mice, 6–8 weeks, 20–25 g; *n* = 6/group	*Lactiplantibacillus/Lactobacillus pentosus* GMNL‐77	Oral gavage; 5 × 10^7^ or 5 × 10^8^ CFU/day	7 days; IMQ applied for 6 days	IMQ + vehicle (distilled water); non‐IMQ control	High‐dose GMNL‐77 reduced erythema, scaling, and epidermal thickness; reduced spleen enlargement	Reduced TNF‐α, IL‐6, IL‐23, IL‐17A, IL‐17F and IL‐22; IL‐17A^+^CD4^+^ and IL‐22^+^CD4^+^ T cells	Not a principal endpoint	High‐dose 5 × 10^8^ CFU/day significantly reduced clinical scores and inflammatory cytokines (*p* < 0.05)
Lu et al., 2021 [45]	Female BALB/c mice, 6–8 weeks, 18–20 g; *n* = 6/group, 10 groups	*Bifidobacterium. adolescentis* CCFM667, *Bifidobacterium. breve* CCFM1078, *Bifidobacterium. animalis* CCFM1148, *Lacticaseibacillus. paracasei* CCFM1074, *Lacticaseibacillus paracasei; Limosilactobacillus reuteri CCFM1147, Limosilactobacillus reuteri* CCFM1132, *Limosilactobacillus reuteri* CCFM1040,	Oral; 5 × 10^9^ CFU/day per strain	Approximately 7 days of probiotic administration with IMQ‐induced disease	Normal control, IMQ control, and methotrexate positive‐control group	CCFM667, CCFM1078, CCFM1074, and CCFM1132 was reported as an effective strain, whereas CCFM1040 was reported as ineffective. Reduced erythema, scaling, thickening, and epidermal hyperplasia; several strains were ineffective	Effective strains suppressed the IL‐23/Th17 axis, including reductions in IL‐23, IL‐17, and/or IL‐22	Effective strains increased SCFAs; several reduced Rikenellaceae; strain‐specific microbiome changes were observed	Significant reductions in selected inflammatory cytokines were reported for effective strains; effects were strain‐specific
Chen et al., 2023 [46]	Female BALB/c mice, 6 weeks, 16–18 g; *n* = 8/group, 8 groups	*Bifidobacterium breve* CCFM683	Oral gavage; 10^6^, 10^7^, 10^8^, 10^9^, or 10^10^ CFU/day	20‐day experiment; IMQ applied during days 15–20	Normal control, IMQ control, and methotrexate positive control	10^9^ and 10^10^ CFU/day reduced ear thickness, cumulative psoriasis/PASI‐like score and histopathological score	10^9^ and 10^10^ CFU/day reduced IL‐17, TNF‐α, IL‐1β, and IL‐6 and modulated keratinocyte/barrier markers	Improved gut microbiota composition/diversity and altered bile‐acid metabolism; effects involved FXR/NF‐κB signaling	Ear thickness, cumulative score, and histopathology: *p* < 0.05 at 10^9^ and 10^10^ CFU/day; lower doses did not show significant clinical/histological benefit

*Note:* IL‐6: Interleukin‐6, IL‐23: Interleukin‐23, IL‐17A: Interleukin‐17A, IL‐17F: Interleukin‐17F, IL‐22: Interleukin‐22, IMQ: imiquimod, BALB/c mice: laboratory mouse strain, IL‐17: Interleukin‐17, and LPS: lipopolysaccharide.

Abbreviations: CD4+ T‐cells, CD4‐positive T‐cells; FXR, Farnesoid X receptor; NF‐κB, nuclear factor kappa B; SCFAs, short‐chain fatty acids; TNF‐α, tumor necrosis factor alpha.

### 4.5. Biological Effects of Orally Administered Immunomodulatory Probiotics in Psoriasis

#### 4.5.1. Effects on Skin Manifestations

Although the probiotics evaluated in the clinical and experimental studies were predominantly administered orally, several studies reported improvements in psoriasis‐related skin manifestations, including reductions in disease severity and inflammatory skin changes. In clinical studies, oral probiotic supplementation has been associated with improvements in PASI and quality‐of‐life measures and reductions in inflammatory biomarkers such as IL‐6 and TNF‐α [38, 39, 42]. Similarly, experimental studies using orally administered probiotics in IMQ‐induced psoriasis‐like models demonstrated attenuation of erythema and scaling, together with reductions in inflammatory cytokines associated with the IL‐23/Th17 pathway [44–46].

These findings suggest that orally administered probiotics may influence cutaneous inflammation indirectly through modulation of the gut microbiota, microbial metabolites, intestinal barrier function, and systemic immune responses. In an experimental model, orally administered *Bifidobacterium* breve CCFM683 was reported to improve psoriasis‐like features while modulating keratinocyte‐associated markers, epidermal barrier proteins, bile acid production, and gut microbiota composition [46]. Thus, skin‐related outcomes may represent downstream consequences of systemic and gut‐mediated immunomodulation rather than direct topical effects.

#### 4.5.2. Systemic Immunomodulatory Effects

Orally administered immunomodulatory probiotics may also exert systemic effects by influencing immune‐cell activity and inflammatory mediators [47, 48]. Proposed mechanisms include modulation of dendritic‐cell function, regulation of Treg/Th17 balance, increased production of anti‐inflammatory cytokine such as IL‐10 and TGF‐β, and reduction of proinflammatory mediators including TNF‐α and IL‐17 [41, 49–51]. The gut microbiota and its metabolites, particularly SCFAs, may contribute to these effects by influencing intestinal barrier integrity and immune‐cell differentiation.

Clinical evidence further supports the potential for orally administered probiotics to modify systemic inflammatory status in patients with psoriasis. Probiotic supplementation has been associated with reductions in circulating inflammatory biomarkers, including CRP, IL‐6, and TNF‐α, although clinical responses have not been consistent across all trials [38–41]. Experimental studies similarly suggest that oral probiotics can modulate the IL‐23/Th17 inflammatory pathway and alter gut microbial composition in psoriasis‐like models [44–46].

Compared with previous systematic reviews and meta‐analyses, the present review provides a broader mechanistic interpretation of probiotic effects in psoriasis. Previous quantitative reviews have primarily focused on determining whether probiotic supplementation improves clinical outcomes such as PASI and DLQI and on summarizing selected inflammatory biomarkers. In contrast, the present review integrates clinical findings with strain‐specific experimental evidence to examine the biological pathways that may connect intestinal microbial modulation with cutaneous inflammation. Particular attention was given to SCFA production, intestinal barrier integrity, dendritic‐cell signaling, and Treg/Th17 homeostasis. Furthermore, the inclusion of strain‐specific experimental studies provides biological context for the heterogeneous clinical findings and may help explain why different probiotic formulations do not produce uniform effects. Thus, the principal contribution of this review is not another summary estimate of probiotic efficacy, but an integrated interpretation of strain‐specific biological mechanisms and their relationship to clinical manifestations of psoriasis.

## 5. Conclusion

This review highlights the potential relevance of the gut–skin–immune axis to psoriasis and summarizes how gut microbiota dysbiosis, intestinal barrier dysfunction, microbial metabolites, and immune signaling may contribute to psoriasis‐associated inflammation. Probiotics may influence these pathways through strain‐specific effects on microbial composition, intestinal barrier integrity, immune‐cell regulation, and inflammatory signaling, including the Treg/Th17 and IL‐23/IL‐17 pathways. However, the current clinical evidence remains limited and heterogeneous, with substantial variation in probiotic strains, formulations, doses, treatment durations, concomitant therapies, and clinical endpoints. The use of combined probiotic–prebiotic interventions in some studies also limits attribution of observed effects specifically to probiotics. Moreover, mechanistic findings from experimental models and other immune‐mediated diseases provide biological plausibility but should not be interpreted as direct evidence of clinical efficacy in psoriasis. Accordingly, probiotics may represent a potential investigational adjunctive approach in psoriasis rather than an established adjunctive treatment. Future research should prioritize adequately powered randomized controlled trials using well‐characterized and standardized probiotic strains and formulations, clearly defined clinical endpoints, appropriate control groups, and sufficiently long follow‐up to establish strain‐specific efficacy, mechanisms of action, and long‐term safety.

## Author Contributions

Ahmed Hussein Hasan Alshihmani, Mahmoud Mahmoudi, Afsane Fadaee, Maryam Ahmadi‐Khorram, and Mohammad Hossein Shams participated in data collection and manuscript writing. Seyed‐Alireza Esmaeili and Ramiar Kamal Kheder designed and drafted the article.

## Funding

The authors have nothing to report.

## Disclosure

All authors have fully read and approved the final manuscript.

## Ethics Statement

The current study is a review article, and the manuscript complies with the ethical rules applicable for this journal.

## Conflicts of Interest

The authors declare no conflicts of interest.

## Data Availability

The data that support the findings of this study are available from the corresponding author upon reasonable request.
